# Micro-Space Complexity and Context in the Space-Time Variation in Enteric Disease Risk for Three Informal Settlements of Port au Prince, Haiti

**DOI:** 10.3390/ijerph16050807

**Published:** 2019-03-05

**Authors:** Andrew Curtis, Robert Squires, Vanessa Rouzier, Jean William Pape, Jayakrishnan Ajayakumar, Sandra Bempah, Meer Taifur Alam, Md. Mahbubul Alam, Mohammed H. Rashid, Afsar Ali, John Glenn Morris, Jr

**Affiliations:** 1 GIS Health & Hazards Lab, Kent State University, Kent, OH 44242, USA; rsquire2@kent.edu (R.S.); jajayaku@kent.edu (J.A.); sbempah@kent.edu (S.B.); 2 Les Centres Haitian Group for the Study of Kaposi’s Sarcoma and Opportunistic Infections (GHESKIO), Port-au-Prince, Haiti; vrouzier@gheskio.org (V.R.); jwpape@gheskio.org (J.W.P.); 3 Emerging Pathogens Institute and Department of Medicine, College of Medicine, University of Florida, Gainesville, FL 32601, USA; mtalam@epi.ufl.edu (M.T.A.); md.alam@epi.ufl.edu (M.M.A.); mhrashid@epi.ufl.edu (M.H.R.); afsarali@epi.ufl.edu (A.A.); jgmorris@epi.ufl.edu (J.G.M.J.); 4 Emerging Pathogens Institute and Department of Environmental & Global Health, College of Public Health and Health Professions, University of Florida, Gainesville, FL 32601, USA

**Keywords:** cholera, informal settlements, GIS, spatial video, Haiti

## Abstract

Diffusion of cholera and other diarrheal diseases in an informal settlement is a product of multiple behavioral, environmental and spatial risk factors. One of the most important components is the spatial interconnections among water points, drainage ditches, toilets and the intervening environment. This risk is also longitudinal and variable as water points fluctuate in relation to bacterial contamination. In this paper we consider part of this micro space complexity for three informal settlements in Port au Prince, Haiti. We expand on more typical epidemiological analysis of fecal coliforms at water points, drainage ditches and ocean sites by considering the importance of single point location fluctuation coupled with recording micro-space environmental conditions around each sample site. Results show that spatial variation in enteric disease risk occurs within neighborhoods, and that while certain trends are evident, the degree of individual site fluctuation should question the utility of both cross-sectional and more aggregate analysis. Various factors increase the counts of fecal coliform present, including the type of water point, how water was stored at that water point, and the proximity of the water point to local drainage. Some locations fluctuated considerably between being safe and unsafe on a monthly basis. Next steps to form a more comprehensive contextualized understanding of enteric disease risk in these environments should include the addition of behavioral factors and local insight.

## 1. Introduction

Monitoring the water supply for diarrheal pathogens in informal settlements (IS) is imperative to improve the health of residents while also being imperative in achieving the targets set out in the United Nations’ Millennium Development Goals [1,2,3,4]. From the research perspective, the need is to understand how, why and when water contamination occurs [5]. While traditional epidemiological data analysis will always be important, this is a complex water-risk landscape comprised of spatial, temporal and behavioral factors that might not so easily be reduced for analysis. Which water points become contaminated and when does this occur, and then how does the environmental configuration, meteorological changes and human action lead to this fluctuation are all important questions. A more nuanced contextualized reading of this complex landscape could lead to both improved daily outcomes, and the ability to respond in the face of a major outbreak. Basic questions that need to be asked include which water points are the most vulnerable to contamination, and does this risk change over time? Even supposedly official “clean” city water piped underground can become contaminated through human activities, such as illegal tapping [6], while reservoirs, irrespective of the cleanliness of the water supply, can become contaminated by having dirty containers lowered into them. Emerging cases of cholera and diarrheal diseases are not only point-in-space outcomes, but also inputs into a complex interconnected system of water access, sanitation and drainage. While conceptually we understand these relationships, we are impeded by a lack of data that reflect the temporally dynamic and spatially granular complexity of the Water, Sanitation, and Hygiene (WASH) [7,8,9] landscape within an informal settlement. Simply put, case locations, water point and drainage testing, fine scale environmental layers, and localized metrological data are all important, but all pose considerable logistical challenges in collection at the scale of intervention, meaning at a granular scale that can support effective community or household intervention. Other contributing factors such as human intra settlement mobility, activity space and context are similarly absent. The goal then is to develop an understanding that includes dynamic epidemiological data collection nuanced by behavioral and/or environmental influences. Ideally, regular water point testing should be supplemented with surveys that capture localized environmental change. Data to be collected would be the setting of each water point with regards to local drainage, standing water and mud, and the condition of the ground at the actual water access point. In this paper, we advance this perspective by focusing on the longitudinal testing of water points for fecal coliforms and *V. cholerae*, with a simultaneous micro-environmental survey. Our aim is to not just analyze spatial and temporal patterns in fecal coliforms but to also develop one of the first contextualized readings of this complex epidemiological, environmental and behavioral system. Our study locations are a coastal informal settlement (IS) in Port au Prince, Haiti. 

The geographic pattern of enteric diseases in an IS is related to the fecal-oral means of transmission [10]. Simply put, household water supplies become contaminated with a diarrheal pathogen. The way this happens is complex and multi-scalar. At the coarsest scale of impact climate change leading to sea level rise, heat/increased water temperature, and rainfall can lead to outbreaks of diarrheal diseases, including cholera [11,12]. However, these broader externalities *work* through localized environmental configurations. For example, flooding or overland flow from drainage ditches containing waste water might contaminate clean water reservoirs during rainy seasons or after intense rain events (such as a hurricane) [13,14,15], but how that water is stored and accessed either protects against or facilitates that contamination. In both Peru and Bangladesh, during strong El Nino years, cholera rates rose in sync with local sea surface temperature [16,17]. In Ecuador, diarrhea rates were found to be positively correlated with seasonal extreme rainfall events [18]. More specifically for this paper, hospital admissions for diarrhea-related illnesses peak in association with seasonal increases in precipitation for the informal settlements of Port au Prince [19]. Yet an IS is not a homogenous space; even with such externalities there is localized variation in disease risk. *Where* is the water point located (does the surrounding relief contribute to area flooding), *how* is the water dispensed (is the source a container sunk into the ground with no seal or other barrier), *how* close are drainage channels or toilets, and is there standing water or mud around the water point where people rest their water containers? Adding further complexity is that these risks are also temporal, as water and mud from cumulative rain events can create a breeding ground suitable for propagating pathogenic bacteria [20]. It is therefore important to develop a more nuanced appreciation of this space in addition to more traditional hotspot and pattern analyses. 

In this paper we explore the interlinked pattern of water point fecal coliform contamination and micro environments for IS neighborhoods in Port-au-Prince, Haiti, that were originally refugee camps after the earthquake of 2010 [21]. In times of such upheaval enteric disease often occurs, and while the current cholera situation in Haiti (as of 2018) is not as severe as that in Yemen where deteriorating hygiene, sanitation systems and water shortages are contributing to a massive human toll [22,23,24], parallels can be drawn due to the human displacement and disruption to clean water and effective sanitation practices that occurred after the earthquake [25,26]. Cholera is now endemic in the region, and knowing how and where disease risk is greatest, especially within the Port au Prince IS, can help spatially prioritize intervention during future outbreaks [27].

### Background to the Study Area

In response to the post-earthquake cholera epidemic, in 2012 Haitian Group for the Study of Kaposi’s Sarcoma and Opportunistic Infections (GHESKIO) organized a cholera vaccination campaign that covered 70,000 people in six IS of Port au Prince [28] (The work described in this paper is part of a partnership with the University of Florida Emerging Pathogens Institute in Gainesville, Florida and GHESKIO which is one of the largest non-profit healthcare providers in the Caribbean. GHESKIO was founded in 1982 in response to the AIDS epidemic, and is now the largest provider of HIV/AIDS and tuberculosis (TB) services in the Caribbean. GHESKIO set up a “tent city” to house over 7000 refugees, many from the three neighborhoods (for ease of description referred to as EA, EC and EB), after the earthquake in 2010, providing shelter, security, food, clean water, health services and educational opportunities. GHESKIO has continued to provide health and social services to the study neighborhoods, and has also trained over 800 community members as “Health Agents” to improve sanitation and access to clean water, and to survey for infectious diseases, malnutrition, and to refer patients to the GHESKIO clinic. During the cholera epidemic the group established an emergency cholera treatment center, and developed comprehensive disease reduction strategies including providing chlorinated water, building of latrines, and establishing rehydration posts, a 250-bed tent hospital, and 10 community health posts). Two of these communities, along with a third unvaccinated neighborhood, are the setting of the study described here. Each of the three study areas are coastal, peri-urban, and to some degree have been reclaimed from the sea through the process of trash dumping and compaction. Each neighborhood also has uneven access to clean water and sanitation systems [19]. The environmental and coliform bacterial data collection described in this paper is part of an initiative to compare and contrast the long-term effectiveness of the original vaccination campaign, while at the same time providing a more detailed understanding of ongoing diarrheal disease risk in the neighborhoods [19]. 

**Figure 1:**
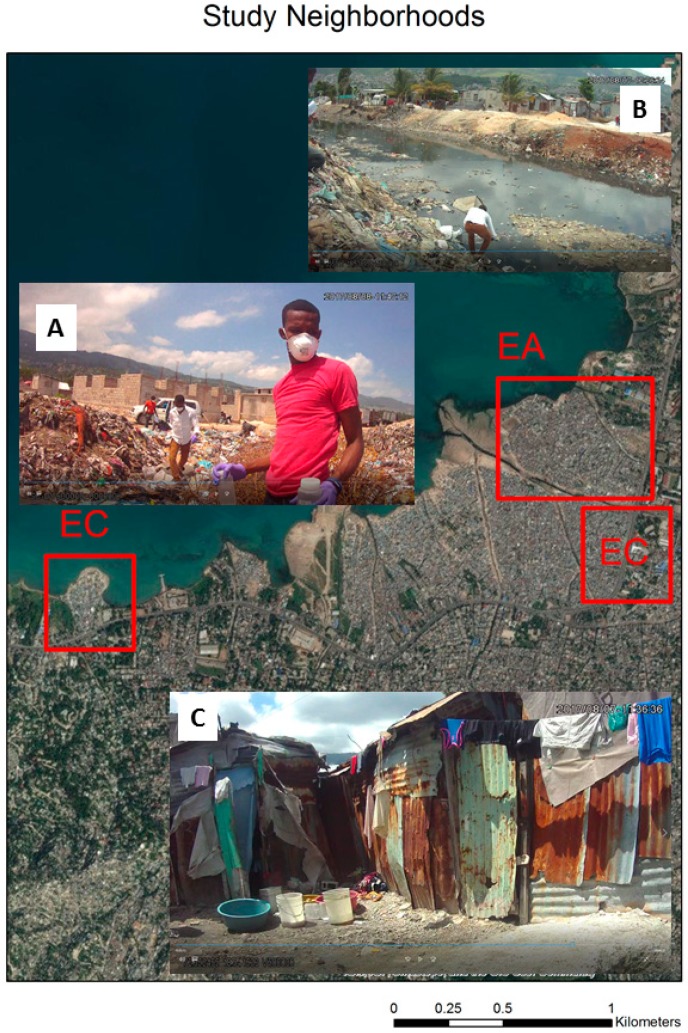
Examples from the three study neighborhoods. (**A**) study neighborhood EA; (**B**) study neighborhood EB; (**C**) study neighborhood EC.

The three neighborhoods have “typical” informal settlements characteristics for the region with structures ranging from concrete block buildings to homes made of corrugated metal and other scavenged materials (Figure 1). Transportation paths are mostly dirt streets or narrow winding alleys. There is virtually no legal built environment infrastructure (such as sewage disposal, electricity, piped water, trash collection), while water is purchased from vendors at a variety of different access points.

Figure 1 contains three inset images from the study neighborhoods named as EA, EC, EB; 1A shows the coastal area EC with the field team walking through accumulated trash piles which often contain fecal matter to take an ocean sample. 1B is water testing in the main drainage channel that separates the neighborhoods EA and EB. The buildings in the background of 1B (located in EC) are a combination of brick/concrete and metal sheet buildings while 1C displays a typical residence found in neighborhood EA. It should be noted that the purpose of this paper is not to focus on the neighborhoods as different units, indeed EA and EB are only separated by the large Bois de Chêne canal. The naming of EA and EB mirrors local operational logistics and is used here for convenience. Instead we treat each testing location by type and as input into multi-scalar complex landscape, one in which variations can occur over relatively small distances. 

**Figure 2:**
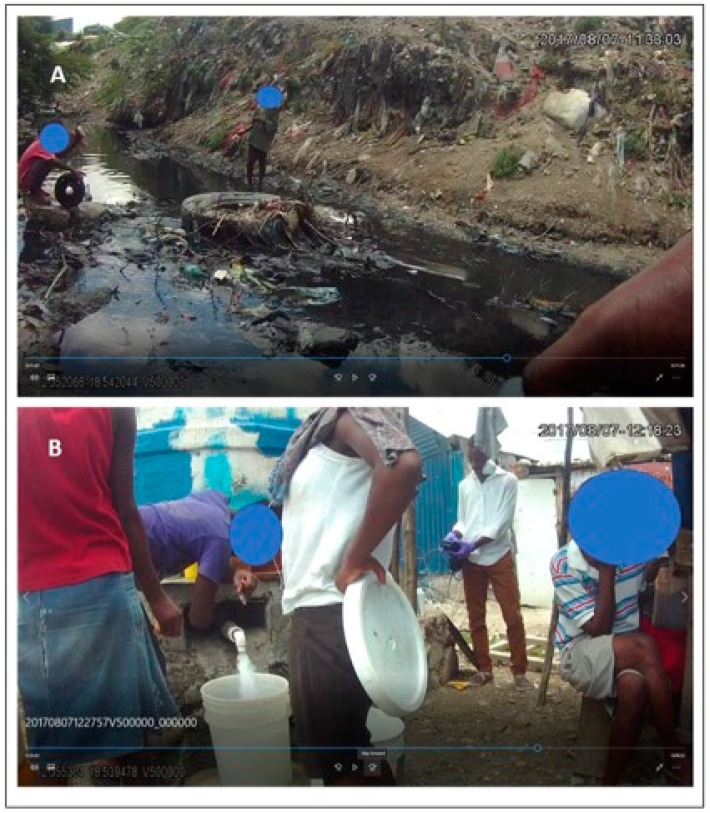
Two example locations of concern; (**A**) is a drainage channel full of trash and waste in which children are playing, and (**B**) is a public water point.

Drainage in the study region is comprised of open channels of varying sizes often full of trash, refuse, human waste and scavenging animals. As can be seen in Figure 2A, which shows one such channel, a risk scenario often described in the literature are children playing in mud or water contaminated with fecal matter [29]. These channels either flow into progressively larger drains, such as the one seen in Figure 1B, or directly into the sea. It should be noted, however, that many of these drains do not have a planned structure to them, but instead are where water flows finding the least resistance. At the sea edge, most notably in neighborhood EC, the land has been pushed out into the water, with the constant dumping of trash forming an unstable surface on which concrete buildings have been built. This can be seen in the background of 1A with new homes being constructed on the trash. The location of this photo is actually several meters into the open ocean on the map of Figure 1 due to the overhead imagery being from 2010.

Figure 2B shows a water point operator filling a customer’s bucket. In the background, one of the project team is testing a water sample taken from the cistern. Water point type varies in the neighborhoods. In Figure 2B an operator distributes from an above-ground reservoir, while elsewhere a concrete cistern or plastic drum reservoir might be set into the ground. For water points without a tap, the customer drops his/her bucket into the storage container, or uses a plastic vessel tethered to the water point. Most of these reservoir-type water points receive water from city trucks. In EC, water points are more frequently stand pipes that have been illegally tapped into the city’s water supply. 

To explore longitudinal patterns of contamination of these water points, we pose the following questions:

- Is there a space-time variation in household water contamination risk, as assessed by fecal coliform (FC) counts, between testing locations?
- Is there a water point type and location that pose the greatest risk to public safety?
- How can micro environmental contextual factors explain the results to the above questions?

## 2. Materials and Methods 

Beginning in October 2016, water samples were collected monthly from thirty-eight locations in three IS neighborhoods of Port au Prince. 

Of eighteen sample sites selected in the EA neighborhoods, ten water points were used for drinking, and/or washing/bathing, one was used for washing/bathing only, four were open drainage channels, and three were ocean collection sites. EB had six sample sites, all of which are a source for drinking and washing water. The EA and EB neighborhoods were only separated by a single large drainage channel. Approximately three kilometers west, the unvaccinated EC neighborhood had fourteen sample locations including six stand pipes illegally connected into the city’s underground water supply, all of which are used for drinking and washing. Five sample locations are drainage channels and the remaining three are from the ocean boundary. 

A team of researchers visited each site to draw water samples which were returned to GHESKIO. Each collected water sample was tested in the field for temperature, turbidity, dissolved solids, pH, and dissolved oxygen, before the sample underwent laboratory testing for FC counts using the membrane fecal coliform (mFC) agar method [15]. Briefly, for the FC assay, we collected 500 mL water in a sterile Nalgene (MilliporeSigma, Burlington, MA, USA) bottle from each sampling site. One hundred mL (100 mL) of directly collected water, and/or, as needed, ten-fold dilutions of the water in phosphate buffered saline (PBS, pH 7.2) [vol/vol] to a final volume of 100 mL were passed through a 0.22 µM filter (MilliporeSigma, Burlington, MA, USA) using vacuum-driven force. The filter was then aseptically transferred onto an mFC agar (MilliporeSigma, Burlington, MA, USA) and the culture plate was incubated overnight at 44.5 ± 0.2 °C. The number of blue bacterial colonies grown on mFC agar were counted and the results were based on the average of triplicate mFC culture plates for each independent water sample tested. mFC culture plates exhibiting no coliform colonies were considered to have <1 cfu/100 mL sample water. As a negative control, an aliquot of 100 mL PBS was processed for FC using mFC agar during each independent experiment.

**Figure 3:**
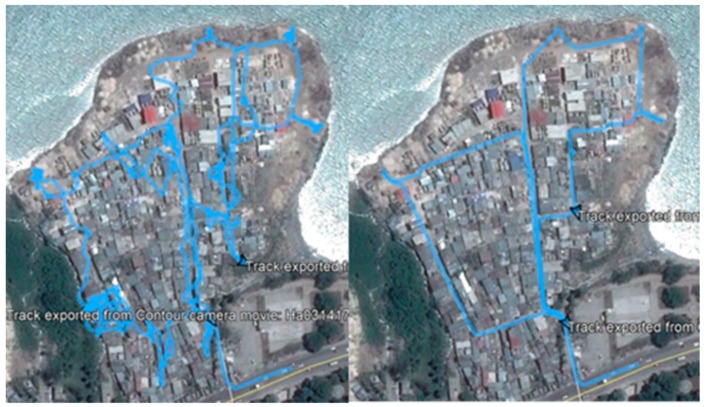
Maps displaying GPS (global positioning system) path corrections in the EC study area. In each image, the blue line shows the walking path connecting test locations. The path on the left is the GPS extracted from the camera. The path on the right has been corrected.

Starting in February 2017, in addition to the water samples, micro environmental surveys were also collected by the field team around each sample site using Contour +2 and Patrol Eye Body cameras. These spatial video (SV) cameras have an internal global positioning system (GPS) receiver, which means the videoed environment around each testing site can be subsequently viewed for risks using CameraPlayer, software the team has developed to simultaneously locate each image frame on a map [30]. After each monthly collection, video and GPS paths were downloaded and meta data sheets completed, which recorded the technical performance of each camera. A combination of the video and overhead aerial imagery was used to verify the accuracy of the GPS path and correct any errors using bespoke software designed by the team for these types of environments [30] (Figure 3). Then, each located video frame became a digitizing source, allowing the team to map “risk” features into Google Earth or as a Geographic Information System (GIS) layer. These risk factors include standing water, mud, trash, and human activity [31,32,33,34]. After the initial SV visit, a map was created for each water point depicting its immediate environmental setting including its proximity to other features such as drainage channels. 

Once an initial environmental assessment had been made of each test location, each subsequent visit was compared for change. This was achieved using an environmental assessment index created with scores ranging from one (least severe risk) to ten (most severe risk) for standing water, mud, trash, and the amount of human activity within a ten meter buffer of the test site. These environmental assessments follow similar methods applied in Haiti, Nicaragua, and Tanzania [30,31,32,33]. This type of mapping of micro-environmental characteristics has been successfully used to track and predict the distribution of both waterborne and water-vectored diseases [35]. While there is always a degree of subjectivity in assigning an environmental score (for example a trash value of 10 for Figure 1A, and 8 for Figure 1B), the same two-person team involving the main coder, and then a project manager overseeing the process with random checks, was used for all sites. This, as much as possible, helped ensure standardization in coding.

The FC counts for each test site, and the associated environmental index were then mapped using ArcGIS 10.4 (ESRI 2011, ArcGIS Desktop, Redlands, CA, USA) and tabulated for exploratory data analysis. 

## 3. Results

### 3.1. Contamination of Neighborhood Water Points

**Figure 4:**
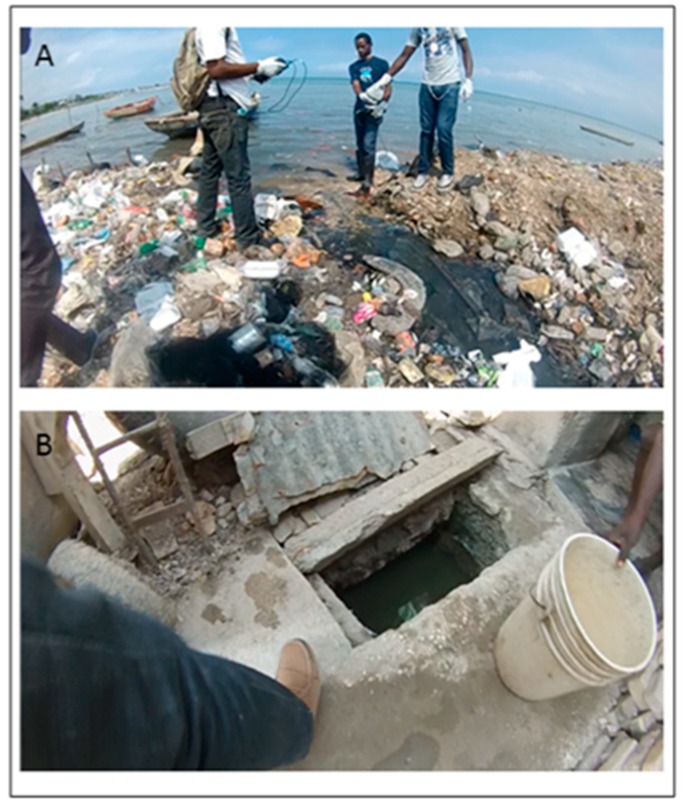
Two environmental testing locations used as a comparison with the household water point samples. (**A**) is an ocean site (EC 11) while (**B**) is a tidal water filled reservoir (EA 16).

Water sampling at thirty-eight test sites began in October 2016 and SV environmental assessments commenced in February 2017. In order to tease out the spatial and temporal variation between sites and the implications these results have for neighborhood residents, the results of the monthly data collection trips are presented in two ways: firstly as a FC comparative table for just household water points and then as a combined FC—environmental assessment. Table 1 displays the results of a ranked FC summary for 22 water points, and two environmental sites (EC 11 and EA 16) which are included for comparative purposes; EC 11 is the juncture of a drainage channel entering the ocean (Figure 4A), and EA 16 is a concrete holding point sourced with ocean water which is also used by the community for household purposes (Figure 4B). All things being equal, it is expected that EC 11 and EA 16 should score the highest FC counts and so they are included as a benchmark of risk by which the other household water points can be judged. Table 1 also includes the water point type and source. 

Table 1 ranks each water point according to its FC count as compared to the other 24 tested locations for that month. Therefore, it expected that EC 11 and EA 16 should score 24 and 23 (the highest possible ranks) for every month. These rankings are also summed for the entire testing period to show the relative stability in risk for each test site. 

**Figure 5:**
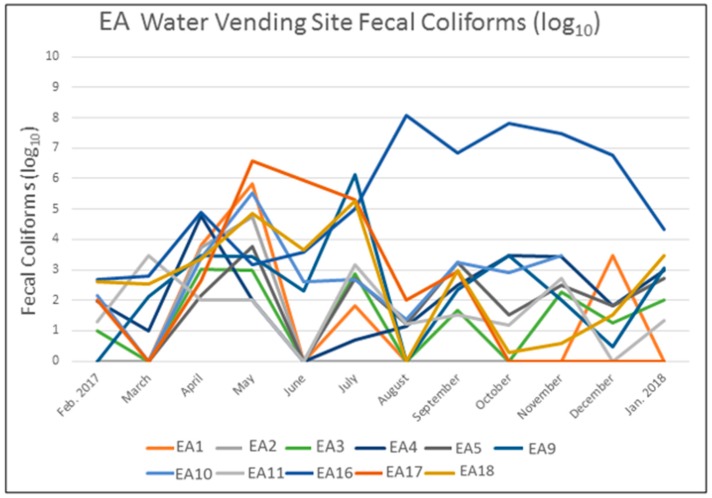
Fecal Coliform counts per month for the water point testing sites in the EA neighborhood.

An alternative way to visualize the risks can be seen in Figure 5 which graphs the FC counts (log transformed) for each of the test sites in the EA neighborhood. No obvious neighborhood-wide pattern is evident, apart from the elevated risk for April and May, which corresponds to the rainy season. However, the same risk is not apparent for the second rainy season (September and October). Indeed, this graph shows that there is considerable fluctuation across all sites, which means that more aggregate “findings” could lose the importance of more localized factors. 

### 3.2. Neighborhood Environmental Assessments

**Figure 6:**
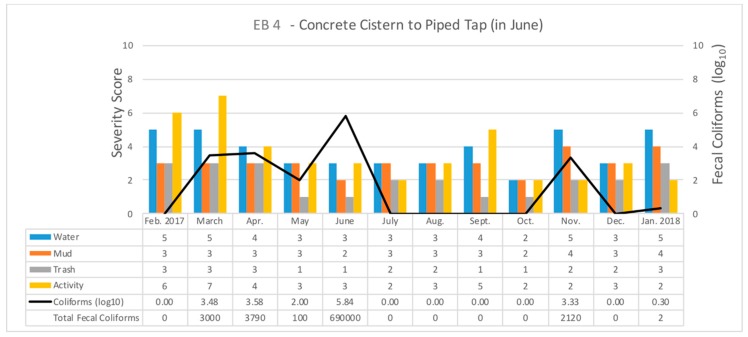
An example of the Fecal Coliform counts and environmental scores for one example water point sample site.

The FC counts were then compared against the environmental contamination scores for each month. Each waterpoint was graphed to include the scores for each contamination category, and the FC count (logged to allow for more easy comparison). Figure 6 provides an example for EB 4.

Using all these outputs, the following profiles were developed for overall IS area. While the EC neighborhood was generally considered safe, the one exception was EC 13 which rose from a low monthly FC count to over 35 million cfu/100 mL (colony forming units per 100 milliliters of collected water) (Table 1). The area around the water point had high levels of standing water and moderately high levels of mud, though not dissimilar from conditions at the other water points in the neighborhood. Indeed, even when FC counts at EC 13 returned to zero the environmental contamination risks persisted for another two months. 

The FC counts in the EB neighborhood were higher than EC, especially EB 1 through 5. Most of these water points are located next to a busy road. Four of the six sites are cistern-style vending points that involve dipping a bucket into the reservoir. Interestingly, over the project period EB 4 changed from a ground-level bucket-retrieval style cistern, to a piped tap by June that connects to the underground city water system. Figure 6 displays the impact of this change with FC counts dropping dramatically by July. The environment around the water point had displayed localized flooding, which would have provided contamination when the cistern was close to ground level, but would have been less problematic when the tap was introduced. FC levels of at least 3000 cfu/100 mL occurred in March and April, before dropping off to 100 in May and then climbing to 690,000 in June when the new tap was introduced. In the months after, until November, levels fell to zero. The spike in June might be explained by the disruption caused by the changeover. 

EB 2 was another water point that experienced a physical change during the study period, as the once jagged and pitted concrete and gravel plinth around the cistern was concreted over. While this permanently reduced the amount of standing water immediately next to the cistern, the potential for contamination remained close by on the sidewalk and roadside which continued to have some of the worst environmental assessment scores in the neighborhood. Interestingly this change had no impact on FC count trends, with a decline from 1350 to 1 from July to August, then rising again to over 2000 in September.

Three of the six collection sites in the EB neighborhood yielded their highest contamination levels in June with EB 1 recording over 1 million cfu/100 mL, EB 4 over 690,000, and EB 6 over 370,000. However, this was not a neighborhood-wide pattern as EB 2, 3, and 5 all reported levels of zero for the same month. Counter to this, each of the EB sites, apart from EB 2, had their lowest environmental assessment scores for June. Indeed, June is tied with May and October as the months of least concern (on average across all six test sites) and is just behind August. Interestingly, October was the only month to have a zero FC count at all the EB household water access points. The most consistently problematic water point was EB 1, a cistern situated at the corner of an extremely busy road and a paved side street, while also being located within 20 m of the Bois de Chêne canal, one of Port-au-Prince’s main open-air sewage canals. EB 1 consistently recorded positive FC results with levels only falling below 50 for four of the months. 

EA generally had the worst water quality. Two of the safer water points, EA 1 and EA 2 were proximate, had a similar water delivery style and cistern structure, and had comparable environmental risks. Both sites saw an FC spike from April to May before becoming relatively safe for the remainder of the study period despite the micro environment around each being consistently wet and muddy. This same FC peak in April and May also occurred in eight of the other eleven water points, with seven of these recording their highest levels during this period (see Figure 6). A secondary FC peak occurred in July which affected six test sites, two of which recording their highest counts. While EA 4 and EA 18 were the most consistently contaminated cisterns (each had only one month with zero FC), their environmental assessments were different; EA 4 generally had proximate water and mud during peak FC months, but EA 18 only had moderate levels of environmental risk coinciding with high FC counts. Possibly a more concerning environmental risk factor for EA 18 was that it had one of the highest human activity measures and is located next to the previously mentioned Bois de Chêne canal. EA 4 and EA 5 were the only water points that displayed a connection between FC counts and their immediate environmental risks. In addition to FC, we isolated two *V. cholerae* nontoxigenic O1 strains from EA 10 and EA 14 in December 2017. 

### 3.3. Environmental Contamination Risk

Eight drainage channels and six coastline/ocean sites were tested in the EC and EA neighborhoods. As one would expect, these sample sites had the highest concentrations of FC. 

#### 3.3.1. Drainage Channels

In general, for the EC neighborhood while there was a consistently high FC count across the months; August saw levels rise for all sites. Even though this was followed by a slight fall, counts remained in the tens of millions through the rest of the study period. There was a consistent level of standing water and mud around each of the EC drainage sites, though the amount of trash varied markedly, being especially high around EC 11, 12, and 14. Some of the sites had visible feces, large amounts of trash and high levels of mud and standing water around the channel. Each drainage channel in the EC neighborhood recorded high FC levels throughout the study period. The source of water into these channels was mostly household runoff (probably also containing some human waste) and rainfall flow. EC 9 was a smaller channel that flowed across a minor road in the southern portion of EC. This flow (at least visibly) appeared to be superficial and serving a local drainage purpose rather than being a collection point of sewage from the broader neighborhood. The environmental scores were lower compared to the other EC drainage sites, though people were often sitting immediately next to the water which flowed under/next to a residence. As an indication of the heterogeneity of risk in the neighborhood, while EC 9 had the lowest FC counts, EC 8 just 15 m away, had the highest FC levels. Indeed, when compared to all neighborhood drainage channels in the EA and EC neighborhood, EC 8 ranked as the fourth most contaminated while EC 9 was the least. 

Four EA drainage sites displayed similar patterns to the EC locations but were notably more severe. EA 12, 13, and 14 were the first, second, and third most contaminated drainage channels in all the study. Interestingly, EA 15, is the previously mentioned Bois de Chêne canal, which flows for several kilometers through Port-au-Prince serving as a catch-all drainage channel, yet it only ranked sixth in terms of FC counts. The canal, which appears to have numerous environmental risks, being full of trash, visible sewage, and feeding pigs, is approximately 15 m wide where the EA 15 samples were taken. Yet while environmental conditions at EA 15 were among the visibly worst at any site, and though FC counts were still high, the less visibly concerning and more proximate to residences EA 12, 13, and 14 drainage samples consistently scored higher.

#### 3.3.2. Ocean Sites

The three EC ocean sites had lower FC counts than those recorded for the drainage channels (presumably because of sea water dilution) though they still remained consistently high across all months. Just as with the drainage channels, FC peaked during August, though differing in hen returning to a pre-peak level. The only exception was EC 2 which had a second peak in December, similar to those also found in the drainage channels. The closest tested drainage (EC 11) was nearly 200 m away, so it is unclear why this secondary spike occurred here but not at the other EC ocean sites. Environmental assessments for the EC ocean sites were somewhat meaningless except for the extraordinary amounts of trash and feces found at the ocean edge.

While the EA drainage sites had higher FC counts than their EC counterparts, the reverse was true for the ocean samples drawn in both neighborhoods. Just as with the EC sites, the land edge to these sample locations had excessive amounts of trash. However, while EA 6 spiked in August, similar to the EC ocean sites, EA 7 had several minor peaks and EA 8 had no single elevated month, though again, as expected, all three maintained generally high FC levels throughout the study period. 

## 4. Discussion

It is widely acknowledged that residents of informal settlements suffer elevated exposure to disease, and that much of this risk is a result of clean water access and sanitation systems. Conceptually, we also understand that this risk is not homogenous but rather a localized function of water point type, drainage, nearby environmental risks, and behavior. In this paper we have attempted to take the first steps in documenting this geographic complexity using monthly FC counts and environmental assessments. Interpretation of these results led to the conclusion that there is a broad difference between the neighborhoods with EC being far safer than EA or EB. Interestingly EC is also the neighborhood that did not receive the cholera vaccine though, obviously the FC counts reported here are not indicative of a specific disease, rather a general level of risk. One explanatory factor for better results in EC was the method of water delivery, which was illegally sourced taps. For the other neighborhoods, where the water was often supplied from a reservoir at or close to ground level, contamination could occur through overland flooding or generally wetter conditions around the water point. This was especially true if the customer used either a tethered container or their own bucket to dip into the reservoir. In these cases, contamination could occur through human contact or by setting the vessel down in pathogen rich water or mud. While these were the general patterns, nuance and variation resulted in a heterogenous surface of risk within each neighborhood. For example, two water points built around a plastic in-ground drum below a city water pipe (EA 1 & 2) were relatively FC free for most of the study period, while the next closest site to these, at 70 m away (EA 18), recorded constant contamination. However, the proximate environmental conditions around EA 18 were visibly better than for EA 1 and 2. 

In another example, while the results in EC suggest a tap is the safest method of access, and with the one EB site improving its water quality after switching to this method further supporting this, there were still taps in the EA neighborhood that recorded months with a high FC. This suggests that other factors, especially the method of water source and storage, still play an important role in contamination. Similarly, proximate environmental risks alone do not predict contamination. Just as with the EA 1 and 2 comparisons with EA 18, the EC neighborhood generally had poor environmental conditions around each water point. This suggests that even if the conditions for potential contamination exist, protective factors (delivery type), and enhancing factors (nearby drainage) will create a heterogeneous intra neighborhood risk map. 

**Figure 7:**
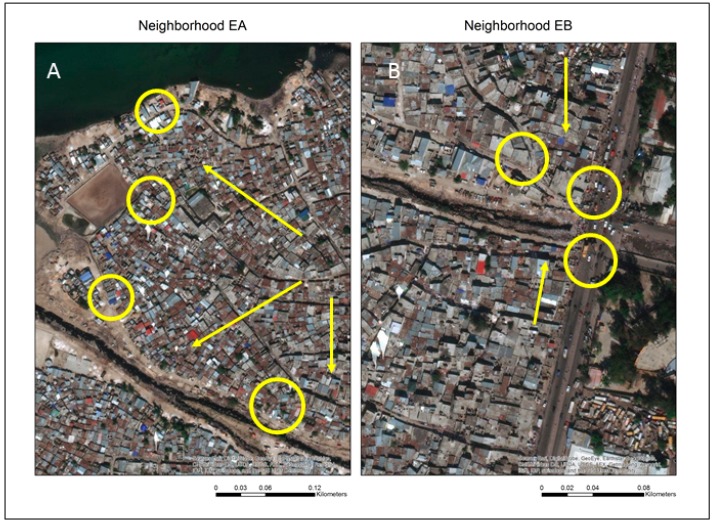
Generalized locations of high risk sample sites in the EA and EB neighborhoods. Arrows indicate the increases in contamination the closer the site is to either the Bois de Chêne canal (**A** and **B**) or the coastal edge (**A**).

There are still more general observations that hold relevance for all study locations. For example, Figure 7 maps all water points in the highest risk category of Table 1 as yellow circles. Two potential spatial patterns emerge, the proximity to the main drainage channel (EA 11, 16, 17, 18 and EB 1), and being at the edge of the neighborhood drainage pattern where water channels reach the sea. This geographic pattern is further supported by EA 4 and 5 which occupy the next two places in the ranking behind the top risk grouping. This map suggests a problematic link when a water point is close to a drainage channel; indeed, each of the top four ranked sites are within 40 m of one another. 

While proximity to a drainage channel should be considered a risk, this relationship does not correlate with the size or appearance of the channel. While there was a distance decay effect of contamination increasing the closer a water point is to the largest and most visibly problematic Bois de Chêne canal, especially in EB, there were smaller drainage channels that ran through the built-up areas that had even higher FC counts. One possible reason might be that the increased flow in the larger channel leads to FC dilution. Irrespective, the fact that interior drainage channels proximate to both homes and water access points had such high FC counts is alarming, especially for those nearby water points sitting in standing water and mud. Future research should certainly explore this water point—drainage system nexus further.

Another location of immediate concern is EA 16, which is sourced by ground-filtered ocean water. The high FC counts recorded here might not raise alarm because water from this location is only used for cleaning and bathing; however when using this as a benchmark, other nearby water points serving household needs had higher FC counts in March, May, June, and August. It is worth re-emphasizing this point—water points where community members would go to purchase household water actually had higher FC counts than a location sourced by the ocean. Adding further temporal complexity, the EA 16 highest FC count occurred between September and December which was when the majority of the other sites experienced their lowest levels.

This last example also illustrates the danger in relying on a cross sectional FC and environmental assessment. While certain water points were continuously problematic, others fluctuated in risk level by the month of study (for example as seen in Figure 5). Part of the explanation for this temporal variation was meteorological impacts, as generally there are patterns of increased FC counts during the rainy season. The mechanism of contamination here is that overland flow from the drainage ditches either directly comes into contact with the water reservoir or the water/mud around the water point. This pattern is supported in the EC neighborhood which showed no such monthly pattern because there is less likely to be contamination from drainage to a tapped water supply. However, the generally wet and muddy conditions in this neighborhood mean that the same contamination pathways exist and might lead to either subsequent risks with transportation or storage in the home. It should also be noted that while meteorological conditions are not likely to vary within the neighborhoods, or even significantly across the three IS, the localized environmental conditions will lead to different factors increasing or ameliorating that risk. This helps explain the variability seen in Figure 5, though other human-caused changes, such as seen in Figure 6, can also change these temporal patterns. It is for reasons such as these that we need to supplement more traditional epidemiological analysis with a more contextualized understanding of the landscape. 

There are several limitations to the current study that could be addressed in future work. Understanding these micro environmental interactions would obviously benefit from fine scale meteorological data. These types of data (for example weekly rainfall) have proved valuable in recent predictive cholera models for Yemen [36]. Unfortunately, the authors do not know of any suitable fine scale meteorological data for Port au Prince. The team has recently purchased a single weather station to gain some basic rainfall data. Failing this, rain effects can be assessed using proxy measures such as standing water or mud. This leads to the question of how frequently should the environmental surveys be conducted? While we have collected by month, should these be collected every week, or after a single rain event? One challenge to exploring some of these questions in a more controlled manner is the challenging nature of these environments. For example, all data collection in the EA neighborhood ceased since April 2018 because of the ongoing gang violence in the area.

While we have used the SV data to map basic environmental risks, there is always the risk of subjectivity. For this project, and to counter inter rater reliability issues, a single coder with a supervisor overseeing and sample checking all digitizing was used. This has proven successful in other environments. Could the SV data be mined further, such as how high is the water access point from the ground, and therefore could flood waters directly enter the storage container? Are there other concrete surfaces close by that could be used as a staging point to rest buckets? 

While we have considered spatial and temporal variation in FC, supplemented with micro environmental assessments, we have not, as of yet, included human insight into these processes. We do not know the choices involved in selecting a water point, or how families interact with the neighborhood waste water system. We do not know how conditions change at night, or even how a typical day brings the members of a family into contact with other enteric disease-causing environments. For example, as seen in Figure 2, we have visual evidence that children play in the drainage channels that probably contribute to the contamination of the neighborhood water points. Therefore, while spatio temporal variation of water point contamination is vital and should continue, future work should contextualize these findings with behavioral insights. One method which has proven successful in providing such contextualization is spatial video geonarratives (SVG), where local commentaries are added to the SV and then mapped [35,37]. SVG could reveal insights into water point choice, community knowledge on water storage and transport risks, perceived risks when rains occur, and more general daily activity patterns, including where children go to play. 

## 5. Conclusions

Seasonal factors, micro environments around each water point, and proximity to broader drainage patterns should all be considered when determining water point contamination risk. This study has found that the two strongest predictors of water safety are (positive) using a tap to limit environmental contamination, and (negative) being proximate to neighborhood drainage patterns. Even for daily prevention strategies the results presented here suggest where water chlorination is immediately needed, and which water points should be more closely monitored. This insight will not only aid practitioners and epidemiologists working to reduce contamination levels but may also influence local prioritization of vending sites. The same information could be used to help inform community groups as to where their water sources are cleanest, and in so doing place more pressure on local vendors to improve their water points.

While there were no cholera cases in these neighborhoods during the project period, one would expect future outbreaks to contain aspects of the results described here. Of the three neighborhoods, EC appears to be safest even though the generally wetter conditions mean that there is a contamination risk during water transport. This is encouraging as this neighborhood was not part of the initial cholera vaccine strategy. Neighborhood EB would have a varying risk, with one water point (C1) needing immediate attention. The cholera risk would vary within neighborhood EA, with those living closer to the “edge”, meaning being proximate to the main drainage channel or the sea, being most at risk due to overland flow from local drainage channels emerging from the densely settled area where presumably the cases would occur. As a result, several water points would need to be closely monitored (V 9 through V 18). These insights might be extremely useful during an outbreak when resources are limited and time of intervention vital. However, for all neighborhoods, the potential for contamination exists, and while we might advocate for spatial prioritization, these should be in addition to more global risk reduction strategies. 

Finally, SV data also allow for virtual return trips to investigate other disease risks, in effect breaking down research silos. The SV revealed environmental risks connected to other health hazards—drainage channels that flow close to (and in some cases under) houses with people often sitting nearby. Not only is this a concern for water borne disease risk but also as a mosquito breeding ground. Another hazard, trash, which can be used in addition to sewage and standing water as a proxy for leptospirosis [38], varied dramatically across the neighborhood with amounts dramatically rising towards the ocean. These trash piles not only attract a wide range of animals (including stray dogs and presumably rats), but also contain a high amount of human feces. 

Similarly, the SV data could be repurposed for non-health usage. A large part of the EC neighborhood is built upon semi-compacted trash and rubble that has amassed over the past 20 years. Unregulated disposal of refuse and subsequent building has allowed this area to expand into the sea. These reclaimed areas are vulnerable to future liquefaction associated with seismic activity, as well as sea water inundation through sea level rise and storms/hurricanes. Evacuation and other hazard mitigation strategies, along with security, infrastructure, amenity and even education planning could benefit from these spatially linked visuals, especially if collected over time, to make more informed intervention strategies. 

## Figures and Tables

**Table 1:** Monthly Fecal Coliform counts for each of the tested water points.

Sample	Type	OctRank	NovRank	DecRank	JanRank	FebRank	MarchRank	AprilRank	MayRank	JuneRank	JulyRank	AugRank	SepRank	OctRank	TotalRank
EC 11	Drain	**22**	0*	**24**	**24**	**24**	24	24	23	**18**	23	24	24	23	277
EA 16	Concrete, ocean	0*	0	**15**	**23**	**23**	**17**	**23**	**14**	**19**	19	23	23	24	223
EA 10	Cistern	14	18	12	10	**20**	0	**16**	21	**17**	**11**	20	**18**	**20**	197
EB 1	Cistern	0*	0*	**18**	**19**	**22**	**19**	**12**	**18**	24	**17**	21	**20**	0	190
EA 9	Cistern	**21**	**22**	11	10	0	**15**	**17**	**16**	**16**	22	0	**13**	**21**	184
EA 11	Plastic container	**20**	**23**	13	10	17	**19**	**7**	**11**	0	**16**	19	11	18	184
EA 17	Cistern	0*	**20**	**16**	10	**19**	0	**9**	24	23	21	22	**16**	0	180
EA 18	Cistern	0*	0*	**23**	10	**21**	**16**	**14**	**20**	**20**	20	0	**17**	17	178
EA 4	Cistern, truck	15	0	10	18	18	14	**22**	9	0	8	16	**14**	**21**	165
EA 5	Cistern, truck	**18**	**21**	9	10	0*	0	**8**	**17**	0	**12**	18	**19**	19	151
EB 2	Cistern	**19**	**19**	**19**	0	0	**19**	**14**	0*	0	**14**	14	**21**	0	139
EB 4	Concrete, city	**16**	**24**	**17**	9	0	**19**	**18**	9	22	0	0	0	0	134
EB 3	Cistern	0	0	**19**	17	15	**19**	**21**	0*	0	**10**	15	**15**	0	131
EB 5	Cistern	0	0	**19**	**21**	0	**18**	**13**	**11**	0	**18**	17	10	0	127
EB 6	Cistern, city	0*	0	**19**	**21**	0	0	**10**	**15**	21	**15**	0	**22**	0	123
EA 01	Drum, city	13	0	0	**20**	0	0	**20**	22	0	9	0	0	0	84
EA 3	Cistern, truck	17	0	0	0	15	0	**11**	**13**	0	**13**	0	12	0	81
EA 02	Drum, city	0	0	14	0	0	0	**19**	**19**	0	0	0	0	0	52
EC 10	Tap, city	22	0*	8	8	0*	0	0	0*	0*	0	0*	0	0	38
EC 13	Tap, city	0	0*	0	0	0	0	0	0	0	24	0	0	0	24
EC 07	Tap, city	22	0*	0	0	0	0	0	0	0	0	0	0	0	22
EC 05	Tap, city	0	0*	7	6	0	0	0	0	0	0	0	0	0	13
EC 06	Cistern, city	0	0*	6	6	0	0	0	0	0	0	0	0	0	12
EC 01	Tap, city	0	0	0	10	0	0	0	0	0	0	0	0	0	10

Test sites with a 0* indicate no value for that month, either because the water point was not available (for example being locked), it was dry having <1 cfu/100 mL sample water, or there was a problem with the sample. Months exceeding 100 cfu/100 mL. (colony forming units per 100 milliliters of collected water) are colored light grey, those exceeding 1000 are colored dark grey, and those exceeding 100,000 are in light red. EA, EC and EB are the three study neighborhoods.
